# Results After Percutaneous and Arthroscopically Assisted Osteosynthesis of Calcaneal Fractures

**DOI:** 10.1177/1071100720914856

**Published:** 2020-05-15

**Authors:** Wolfram Grün, Marius Molund, Fredrik Nilsen, Are Haukåen Stødle

**Affiliations:** 1 Department of Orthopaedic Surgery, Østfold Hospital, Grålum, Norway; 2 Division of Orthopaedic Surgery, Oslo University Hospital, Oslo, Norway

**Keywords:** calcaneal fracture, subtalar arthroscopy, percutaneous osteosynthesis

## Abstract

**Background:**

Operative treatment of calcaneal fractures using the extensile lateral approach is associated with high rates of soft tissue complications. In the past years, there has been a trend toward less invasive surgical approaches. Percutaneous and arthroscopically assisted calcaneal osteosynthesis (PACO) combines the advantages of visualization of the posterior facet of the subtalar joint with a minimally invasive approach.

**Methods:**

We conducted a follow-up of 25 patients with 26 calcaneal fractures (Sanders II and III), treated with PACO with a minimum follow-up of 12 months. The median age was 44 years (range, 21-72) and the follow-up period 15 months (12-33). Our clinical outcomes were the Manchester-Oxford Foot Questionnaire (MOxFQ), the Calcaneus Fracture Scoring System (CFSS), the American Orthopaedic Foot & Ankle Society (AOFAS) Ankle-Hindfoot score, the Short-Form-36 (SF-36), the visual analog scale (VAS) for pain, and the number of complications. Radiographs on follow-up were obtained to evaluate the reduction of the fractures as well as osteoarthritis of the subtalar joint.

**Results:**

The median MOxFQ score was 26.6 (0-76.6), the CFSS score 85 (26-100), and the AOFAS score 85 (50-100). The VAS pain score was 0 (0-5.7) at rest and 4.1 (0-8.2) during activity. The Böhler angle improved from a mean (SD) of 3.5 (12.3) degrees preoperatively to 27.7 (10.5) degrees postoperatively. The follow-up radiographs showed subsidence of the fractures and a Böhler angle of 20.3 (12.9) degrees. There were no wound-healing complications. Two patients had additional surgery with screw removal due to prominent hardware.

**Conclusion:**

Our results suggest that PACO gives good clinical outcomes and a low risk of complications in selected calcaneal fractures. Prospective long-term studies will be necessary to better document the potential advantages and limitations of this operating technique.

**Level of Evidence:**

Level IV, retrospective case series.

The treatment of displaced intra-articular calcaneal fractures has been a controversy during the past decades. Operative treatment through an extensile lateral approach has been the gold standard despite high rates of infection and soft tissue complications.^1,13,19^ Several randomized controlled trials (RCTs) have raised doubt whether open surgery of displaced intra-articular calcaneal fractures is justified.^2,6,12^ Lately, there has been a trend toward less invasive fixation methods such as mini-invasive plate osteosynthesis using the sinus tarsi approach^3,20,34^ as well as percutaneous techniques.^24,27,28^ In 2002, a percutaneous technique assisted by hindfoot arthroscopy was introduced on Sanders II fractures by Rammelt et al.^23^ Since then, a total of 8 studies that evaluate the results of 108 patients with 111 fractures treated by percutaneous and arthroscopically assisted calcaneal osteosynthesis (PACO) have been published.^11,18,21-23,26,33,35^ These studies have shown promising clinical results and a low rate of complications. However, the indication for PACO and the surgical technique varies between these studies.

We wanted to conduct a retrospective study examining the outcome after operative treatment of selected Sanders II and III calcaneal fractures using the PACO technique. We hypothesized that the clinical scores and patient-related outcome scores were comparable to the outcome after using the extensile lateral approach reported in the literature with minimal infection and soft tissue complication rates.

## Materials and Methods

We have performed PACO since 2013 in selected Sanders II and III fractures at Østfold Hospital and Oslo University Hospital (level 1 trauma center). The indication for using the PACO technique was made by the surgeon, dependent on whether the respective fracture pattern was eligible for this operative treatment or not. Our requirements were a constant fragment that was not comminuted and large enough to provide good purchase of the screws and a tuber fragment large enough to give good purchase of both the Schantz screw during the reduction maneuver and the rafting screws for definitive fixation. In addition, an intra-articular fracture pattern that was reducible with a closed technique (Sanders II and selected Sanders III fractures) was a prerequisite for the procedure.

Our inclusion criteria for the present study were as follows:

- Closed, dislocated intra-articular calcaneal fracture treated with only percutaneous reduction and fixation and assisted by subtalar arthroscopy between 2013 and September 2018
- Age 18 years or older at the time of injury
- Availability for follow-up: We excluded patients who had moved abroad and therefore were unavailable for follow-up and patients who could not participate due to other relevant illness (eg, dementia)

Patients with bilateral fractures, diabetes mellitus, severe neurovascular insufficiency, or critical soft tissue conditions as well as smokers were not excluded from our study. The study was approved by the local ethics committee at Østfold Hospital and Oslo University Hospital.

Thirty patients with 32 calcaneal fractures that were operated upon between October 2014 and September 2018 matched our inclusion criteria. All of them were invited to participate in a follow-up examination with a minimum follow-up period of 12 months. Five patients declined a follow-up examination and were lost to follow-up. This left a total of 25 patients with 26 fractures for clinical and radiographic follow-up, representing a follow-up rate of 83%. All patients signed an informed consent form. Twenty-three men and 2 women with a median age of 44 years (range, 21-72) and a median follow-up of 15 months (12-33) were examined. One patient had bilateral calcaneal fractures. Ten of the 25 patients (40%) were smokers at the time of injury. One patient had diabetes, and none of our patients had neurovascular insufficiency at the time of surgery. There were 17 Sanders II (5 Sanders IIA, 10 Sanders IIB, and 2 Sanders IIC) and 9 Sanders III fractures (7 Sanders IIIAB and 2 Sanders IIIAC) (Table 1). Twenty-three of the 25 patients sustained their fractures by a fall from a height, 1 fell from a downhill mountain bike, and 1 was injured while performing martial arts. Two patients had concomitant injuries: 1 patient sustained a burst fracture of the first lumbar vertebra, which needed operative treatment, and 1 patient sustained a comminuted distal radius fracture that was treated with external fixation.

**Table 1.** Demographic Data of Our Study Population.^a^

Characteristic	Value
Patients	25
Calcaneal fractures	26
Sanders II	17
Sanders III	9
Sex, male/female	23/2
Smokers	10 (40)
Patients with diabetes	1
Patients with neurovascular disease	0
Age at injury^b^ (y)	44 (21-72)
Time injury-surgery^b^ (d)	4 (1-12)
Duration of surgery^b^ (min)	139 (73-234)
Postoperative hospitalization^b^ (d)	3 (1-6)
Total hospitalization^b^ (d)	6 (3-17)
Follow-up^b^ (mo)	15 (12-33)

a Values are presented as number (%) unless otherwise indicated.

b Values are presented as median (range).

### Operative Technique

**Figure 1.**
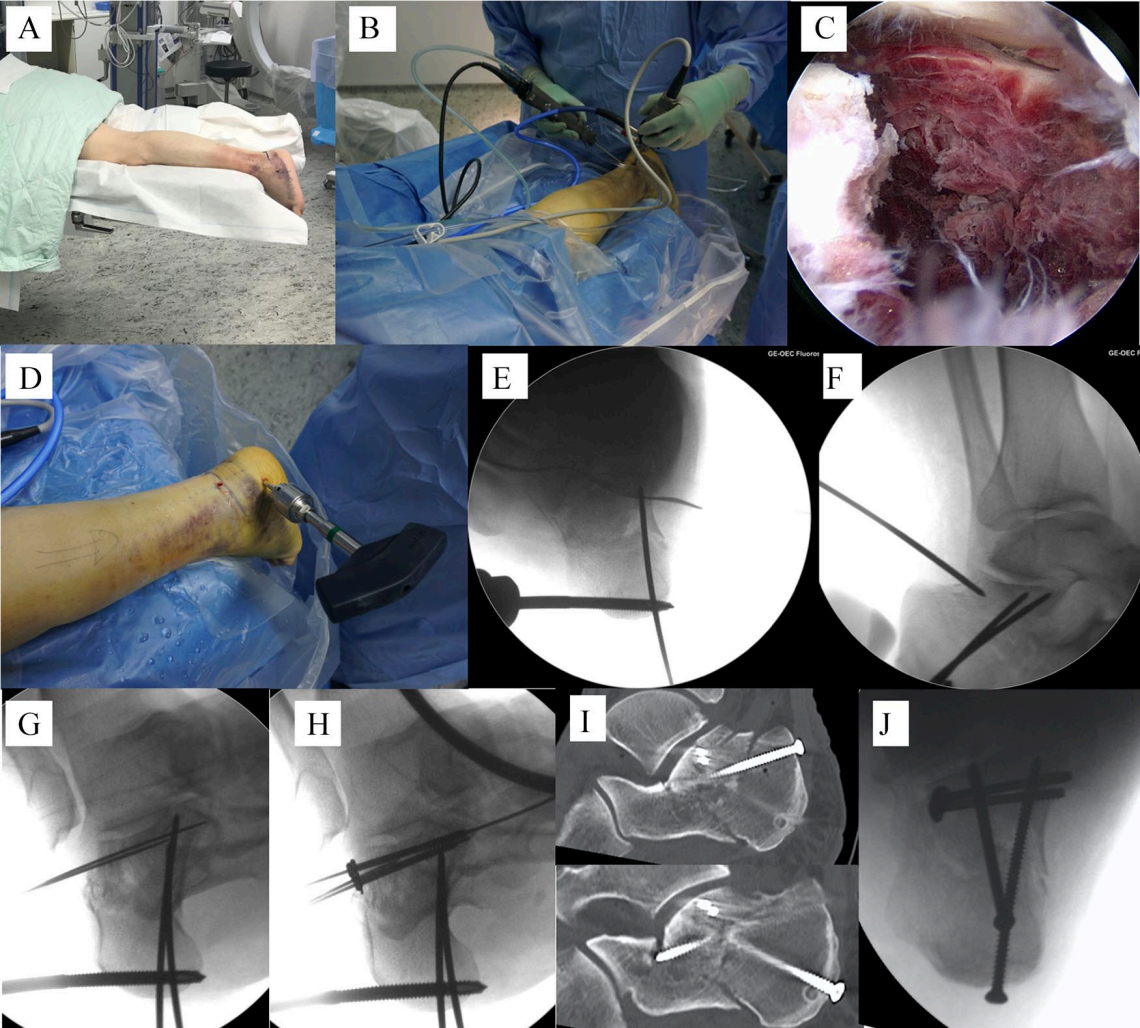
Operative technique. (a) Positioning of the patient. (b) Posterior ankle arthroscopy. (c) Entering the fracture and the subtalar joint. (d) Placing a Schantz screw in the tuber fragment. (e) Reduction of the tuber fragment and temporary fixation. (f) Reduction of the posterior facet fragment(s). (g) Temporary stabilization of the joint fragment(s). (h) Screw fixation of the joint fragments. (i) postoperative sagittal computed tomography scans. (j) Postoperative axial fluoroscopy.

The patient was placed in a prone position (Figure 1a). The uninjured leg was lowered to facilitate fluoroscopy. A thigh tourniquet was used. Standard posterolateral and posteromedial portals for posterior ankle and subtalar arthroscopy were made as introduced by van Dijk et al^29^ (Figure 1b). We use a standard 30-degree, 4.0-mm knee arthroscope and a shaver. The subtalar joint and the fracture fragments were visualized (Figure 1c). Debris and hematoma were removed with the shaver. A 5-mm Schantz screw was placed into the tuberosity fragment and used as a joystick (Figure 1d). The tuber fragment was reduced by first restoring anatomical length and then reducing any varus malalignment. Thereafter, the tuber fragment was temporarily fixated to the constant fragment using K-wires (Figure 1e). It was crucial that both the tuber and the constant fragment were large enough to successfully perform this technique. Care was taken not to place the K-wires too lateral as they then would have interfered with the further reduction of the lateral fragment(s). In the next step, 1 or 2 K-wires that were sharp-ended on both ends were placed from lateral into the fracture surface of the constant fragment and through the skin medially. The wires were then backed out medially under the guidance of the arthroscope, so that the lateral tip of the wires was left flush with the fracture bed of the constant fragment. Then, under arthroscopic visualization, the posterior facet was reduced percutaneously using a periosteal elevator, Steinmann pin, or Schantz screw (Figure 1f). If necessary, an additional sinus tarsi portal was made to improve the visualization. When reduction of the posterior facet was accomplished, it was secured with the previously placed K-wires, which were now advanced from medially into the reduced lateral joint fragment(s) (Figure 1g). To secure the reduction of the articular fragments, two 3.5-mm screws were placed from lateral into the lateral joint fragment(s) and the sustentaculum, using a lag technique. Cannulated screws could be used, as well as washers, to improve fixation. During the screw placement, the K-wires were used as a guide for optimal screw placement (Figure 1h). After the articular fragments were fixed, the tuberosity fragment was fixed with either 3.5-mm or 4.5-mm rafting screws toward the anterior process and/or the constant fragment, giving additional support to the posterior facet and securing the axis and length of the calcaneus (Figure 1I, 1J). Additional screws could be placed to improve stability, dependent on the nature of the fracture.

### Rehabilitation Protocol

Postoperatively, no cast, splint, or brace was applied. Patients were kept nonweightbearing for 8 weeks but were encouraged to do range-of-motion exercises for the ankle and subtalar joint during that time. After 8 weeks, the patients were allowed weightbearing as tolerated using normal shoes. This protocol was applied to all of our patients, including the smokers, the patient with diabetes, and the patient who had bilateral fractures.

### Clinical Examination

At follow-up, all patients were examined by the first author (W.G.). Our clinical outcome scores were the Manchester-Oxford Foot Questionnaire (MOxFQ),^8^ the Calcaneus Fracture Scoring System (CFSS),^15^ the American Orthopaedic Foot & Ankle Society (AOFAS) Ankle-Hindfoot score,^17^ the Short-Form-36 (SF-36),^30^ and the visual analog scale (VAS) for pain. The MOxFQ is a foot and ankle–specific patient-reported outcome measure (PROM) scoring from 0 to 100, with 0 representing the best possible result. The CFSS is a calcaneal fracture–specific scoring system, evaluating pain at rest and on activity, work status, walking ability, and the use of walking aids. This results in a score of 0 to 100, with 100 representing an optimal result. The AOFAS Ankle-Hindfoot score is a widely used score that evaluates pain, function, and alignment, resulting in a score of 0 to 100, with 100 representing an optimal result. The SF-36 is a quality-of-life score, consisting of 8 subgroups that are used to calculate the physical and mental component summary (PCS and MCS). The VAS for pain both at rest and on activity results in a score from 0 to 10, with 0 representing no pain and 10 representing unbearable pain.

Range of motion (ROM) of both the ankle and the subtalar joint was measured using a goniometer and compared to the ROM of the uninjured side. The number of complications and reoperations was determined both through chart review and during the follow-up examination. Furthermore, the time for return to work was recorded.

### Radiological Examination

The radiographic examination consisted of lateral and axial radiographs to evaluate the reduction of the fractures as well as Brodén views to identify the presence of osteoarthritis of the subtalar joint. To reduce bias, 3 of the authors (W.G., F.N., A.H.S.) independently assessed the Böhler angle on the radiographs taken preoperatively, postoperatively, and at follow-up, as well as on the radiographs taken of the uninjured side. Subtalar osteoarthritis was graded with the classification by Kellgren and Lawrence,^14^ which is a proper classification system for subtalar osteoarthritis.^9^

### Statistics

Statistical analysis was performed using SPSS 25.0 statistical software (SPSS, Inc, an IBM Company, Chicago, IL). Parametric data are presented with means and standard deviations while nonparametric data are presented with median and range; *t* tests were used for analysis of parametric data and Wilcoxon tests for analysis of nonparametric data. A *P* value of less than .05 was considered statistically significant.

## Results

### Clinical Results

The median MOxFQ score was 26.6 (0-76.6), the median CFSS was 85 (26-100), and the median AOFAS score was 85 (50-100). The median VAS pain score was 0 (0-5.7) at rest and 4.1 (0-8.2) during activity (Table 2).

**Table 2.** Clinical Results.^a^

Characteristic	Value
MOxFQ	26.6 (0-76.6)
CFSS	85 (26-100)
AOFAS score	85 (50-100)
VAS at rest	0 (0-5.7)
VAS at activity	4.1 (0-8.2)
Ankle ROM, deg	
Operated side	40 (33-90)
Uninjured side	55.5 (36-100)
Subtalar ROM, deg	
Operated side	22 (5-37)
Uninjured side	30 (14-45)
Complications, No.	
Infection or soft tissue complication	0
Temporary nerve affection	4
Additional surgery	2 (screw removal)

Abbreviations: AOFAS, American Orthopaedic Foot & Ankle Society; CFSS, Calcaneus Fracture Scoring System; MOxFQ, Manchester-Oxford Foot Questionnaire; ROM, range of motion; VAS, visual analog scale.

a Values are presented as median (range) unless otherwise indicated.

The results of the SF-36 PROM are shown and illustrated in Table 3, comparing our results to the Norwegian reference values for the male age group 40 to 49 years, which appropriately represents our study population.^10^ Comparing our SF-36 subgroup results with the reference values, we found our study population to have a significantly lower Physical Function (PF) result (*P* < .05). However, we found no statistically significant difference in the other SF-36 subgroups Physical Role (RP), Bodily Pain (BP), General Health (GH), Vitality (VT), Social Function (SF), Emotional Role (RE), and Mental Health (MH), as well as in the PCS and MCS.

**Table 3.** Results of the SF-36 Subgroups.

Subgroup	Study Population, Median (Range)	Norwegian Mean (SD) Reference Values, Men 40-49 Years^10^	*P* Value
PF	85 (15-100)	91.7 (14.6)	.01
RP	100 (0-100)	84.2 (31.0)	.81
BP	72 (22-100)	76.6 (25.0)	.27
GH	87 (30-100)	79.1 (19.7)	.16
VT	70 (20-90)	64.3 (19.4)	.56
SF	100 (12.5-100)	88.4 (19.5)	.46
RE	100 (0-100)	90.4 (25.3)	.17
MH	80 (36-88)	81.1 (15.6)	.21
PCS	51.6 (24.0-58.6)	51.5 (8.3)	.64
MCS	55.9 (26.3-60.7)	53.0 (8.5)	.46

Abbreviations: BP, Bodily Pain; GH, General Health; MCS, Mental Component Summary; MH, Mental Health; PCS, Physical Component Summary; PF, Physical Function; RE, Emotional Role; RP, Physical Role; SD, standard deviation; SF, Social Function; VT, Vitality.

The median ROM of the ankle joint was 40 degrees (33-90) on the operated feet compared to 55.5 degrees (36-100) on the uninjured side (*P* < .01), representing 72% of the ROM of the uninjured joint. The median ROM of the subtalar joint was 22 degrees (5-37) on the operated feet compared to 30 degrees (14-45) on the uninjured side (*P* < .01), representing 73% of the ROM of the uninjured joint.

In the clinical examination, all patients had plantigrade feet without any clinical varus or valgus malalignment of the hindfoot. All but 1 patient was using normal shoes at follow-up. Twenty patients returned to their former occupation at a median 3.25 months (0.5-15), 1 patient had to change occupation for reasons related to the calcaneal fracture, and 2 patients had become incapable of occupational work as a result of their injury. Two patients were already retired when they sustained their calcaneal fracture.

### Radiographic Results

The mean (SD) Böhler angle increased from preoperatively 3.5 (12.3) degrees to 27.7 (10.5) degrees postoperatively (*P* < .001). The follow-up radiographs showed subsidence of the fractures to a mean (SD) Böhler angle of 20.3 (12.9) degrees, representing a statistically significant difference compared to the postoperative Böhler angle (*P* < .001) as well as to the mean Böhler angle of the uninjured side, which was 32.6 (3.8) degrees (*P* < .001). Twenty-five of the operated 26 feet (96%) showed signs of subtalar osteoarthritis. Ten feet (38%) had Kellgren and Lawrence grade 1 osteoarthritis, 9 feet (35%) grade 2, and 6 feet (23%) had grade 3. No patient had grade 4 osteoarthritis (Table 4).

**Table 4.** Radiographic Results.

Characteristic	Value
Böhler angle preoperatively, deg^a^	3.5 (12.3)
Böhler angle postoperatively, deg^a^	27.7 (10.5)
Böhler angle at follow-up, deg^a^	20.3 (12.9)
Böhler angle uninjured side, deg^a^	32.6 (3.8)
Subtalar osteoarthritis (Kellgren and Lawrence), No. (%)	
Grade 0	1 (4)
Grade 1	10 (38)
Grade 2	9 (35)
Grade 3	6 (23)
Grade 4	0 (0)

a Values are presented as mean (standard deviation).

### Complications

There were no wound-healing complications or surgical site infections. Four patients reported temporary nerve problems, which resulted in postoperative numbness in parts of the hindfoot. At final follow-up, all patients had normal sensibility in their operated feet. Two patients had additional surgery with removal of selected screws, 1 due to a prominent screw head and 1 due to irritation of the medial plantar nerve. No other complications were observed.

## Discussion

The results from this study show that percutaneous and arthroscopically assisted osteosynthesis of calcaneal fractures provided good clinical outcomes with a low rate of surgical site infections or wound-healing complications. In the present study, the median MOxFQ score was 26.6 (range, 0-76.6), whereas the median AOFAS score was 85 (50-100). We have not found any other comparable studies using the MOxFQ score, but there are a few studies reporting the AOFAS score after treating Sanders II and III fractures using arthroscopically assisted percutaneous techniques. Sivakumar et al^26^ reported a mean AOFAS score of 87.8 and a mean CFSS of 83.6 after a mean 14 months of follow-up, while Yeap et al^35^ reported an AOFAS score of 86.7 at 17 months of follow-up. The results of the present study correlate with these previous reports confirming good results with the PACO technique. Pastides et al,^21^ on the other hand, reported a mean AOFAS score of 72 and a mean CFSS score of 79.3 at 2-year follow-up. The somewhat poorer results in that study might be explained by the higher proportion of Sanders III fractures as well as the use of a modified AOFAS score, only comprising the subjective components.

Several studies comparing the extensile lateral approach (ELA) with the sinus tarsi approach (STA) in Sanders II and III fractures have been published. Basile et al^4^ evaluated a mean AOFAS score of 82 in both groups after a minimum follow up of 24 months. Weber et al^31^ reported a mean AOFAS score of 83 in the ELA and 87 in the STA group, which corresponds well with the findings of Zhou et al^36^ (mean AOFAS score of 83 in the ELA and 88 in the STA group). The PACO technique seems to provide the same good clinical outcome as reported in the aforementioned studies.

The ELA was reported to give better results than minimally invasive approaches in the RCT by Khurana et al.^16^ The drawbacks of their study are the small number of patients (20) and that 3 different minimally invasive techniques were used among the 11 patients in the minimally invasive group. Only 4 of the patients in this group were treated using a percutaneous technique only. K-wires with or without an additional external fixator were used for fracture fixation in these 4 patients.

We found the Physical Function (PF) subgroup of the SF-36 to be significantly lower than the value of the Norwegian reference population.^10^ None of the other subgroups of the SF-36 as well as the PCS and MCS showed any statistically significant difference when being compared to the reference values. Buzzi et al^7^ obtained similar results to ours with a PCS of 50.1 and an MCS of 55.3 after treating their study population with the extensile lateral approach. Yeap et al^35^ evaluated a PCS of 57.0 and an MCS of 45.6 in their cohort operated by PACO, representing a higher PCS than our study population.

We found a statistically significant decreased ROM of the operated feet compared to the uninjured side, which corresponds to the results reported by Schepers et al^24^ in their study on percutaneous fixation of Sanders II through IV fractures. They reported 10% reduction of sagittal motion and a 30% reduction of subtalar motion compared to the noninjured side.

Lorenz Böhler^5^ originally described a physiological range of the “tuber-joint-angle” between 30 and 35 degrees. More recent studies evaluated a mean (SD) Böhler angle of 36.4 (4.2) degrees in a healthy British study population^32^ and 34 (5) degrees in a Croatian study population.^25^ We found the Böhler angle of the noninjured feet to have a mean (SD) value of 32.6 (3.8) degrees.

After reduction of the fractures, we were able to achieve a correction of the Böhler angle from a mean 3.5 degrees after injury to 27.7 degrees postoperatively. However, we observed a statistically significant subsidence (*P* < .001) of the Böhler angle at follow-up to 20.3 degrees. The subsidence of the Böhler angle has not been reported to the same extent in other PACO studies. Woon et al^33^ observed a mean postoperative Böhler angle of 21.3 degrees with a minimum subsidence to 20.1 degrees at 2 years of follow-up. Law et al^18^ observed a subsidence from 18.7 degrees postoperatively to 17.6 degrees at long-time follow-up. In their study, Sivakumar et al^26^ had a subsidence from 25.0 degrees postoperatively to 23.4 degrees at final follow-up. However, Tantavisut et al^27^ reported a postoperative Böhler angle of 18.1 degrees subsiding to 13.1 degrees at 3 months postoperatively after using a percutaneous technique on 182 feet in 153 patients. Although we report a larger subsidence than the aforementioned studies, it seems that we achieved an immediate postoperative Böhler angle that was closer to the anatomical Böhler angle. The Böhler angle at final follow-up, however, seems to be comparable. The nonsmokers did not have a statistically significant difference in subsidence compared to the smokers and the patient with diabetes. The reported subsidence might be due to suboptimal screw placement and fixation technique, poor patient compliance, and/or a too aggressive rehabilitation protocol. Our patients were permitted full weightbearing 8 weeks after surgery, while Woon et al,^33^ Sivakumar et al,^26^ and Law et al^18^ permitted full weightbearing after 12 weeks. This might contribute to the amount of subsidence of the Böhler angle observed in the present study.

Twenty-five of the 26 operated feet showed signs of subtalar osteoarthritis. None of the previous studies reporting results after using the PACO technique have evaluated the incidence or severity of posttraumatic subtalar osteoarthritis. Buzzi et al^7^ found grade 0 osteoarthritis in 11%, grade 1 in 29%, grade 2 in 29%, grade 3 in 23%, and grade 4 in 7% (mean follow-up of 5.5 years) after treating calcaneal fractures. Their distribution seems to be comparable to ours (Table 4), although none of our patients had grade 4 osteoarthritis. Basile et al^4^ found mild osteoarthritis in only 5.3% of their patients treated with ELA and moderate osteoarthritis in 5.3% of their patients treated with STA after a minimum follow-up of 24 months. Tantavisut et al^27^ treated their patients with a percutaneous screw fixation technique and reported a subtalar arthrodesis rate of 6.5% with a mean follow-up of 2.6 years (range, 1-8.9 years). To our surprise, we found few studies providing detailed reports on posttraumatic subtalar osteoarthritis. Hence, posttraumatic subtalar osteoarthritis might be underreported in many studies.

Few complications have been reported in previously published studies using the percutaneous and arthroscopically assisted technique, consisting of over 100 cases. Of the reported complications, there was 1 seroma that resolved uneventfully at 6 weeks^33^ and 1 case of portal site infection that was successfully managed with oral antibiotics.^21^ Our findings confirm this low rate of wound complications when using a percutaneous technique. Wound complications and infections, on the other hand, are the main concerns using the ELA in treating calcaneal fractures. These complications are reported to occur in up to 32%.^1^ The trend toward using more minimally invasive techniques in treating calcaneal fractures is mainly motivated by the high complication rate using the ELA. A recent meta-analysis of the STA and the ELA showed a wound-healing complication rate of 4.9% in the STA and of 24.9% in the ELA.^20^

We consider PACO to be a promising operative technique for selected calcaneal fractures. It combines the advantages of a percutaneous approach, reducing the complication risk, with the advantages of arthroscopy, providing full visualization of the posterior facet of the subtalar joint. Although we performed surgery on 1 patient as early as 1 day after injury, we consider it advantageous to postpone surgery until 4 to 7 days as this will reduce the bleeding from the fracture site and the fracture will still be easily reducible.

**Figure 2.**
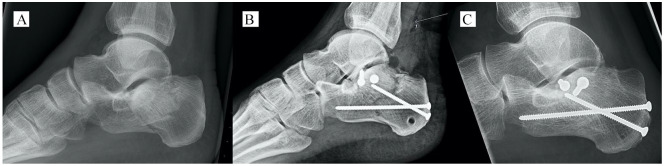
Subsidence of the Böhler angle. A 44-year-old man with a Sanders IIIAB fracture. The Böhler angle was (A) preoperatively –2 degrees, (B) postoperatively 33 degrees, and (C) at follow-up 12 months after surgery 9 degrees. The Manchester-Oxford Foot Questionnaire was 23.4, the Calcaneus Fracture Scoring System was 94, and the American Orthopaedic Foot & Ankle Society score was 87. The amount of subsidence might be explained by the insufficient rafting screw below the subtalar joint.

We recommend starting with selected Sanders II fractures and advise against using PACO on fractures with a comminuted or small sustentaculum fragment to avoid insufficient purchase of the screws that keep the intra-articular fragments reduced. The PACO technique is an advanced and challenging technique; with further experience, we expect to improve our operative technique as well as our results. Based on our findings regarding the subsidence evident from the Böhler angle, we have introduced a more restrictive rehabilitation protocol, not permitting full weightbearing before 10 to 12 weeks postoperatively. As the case in Figure 2 demonstrates, one should aim for optimal placement of the rafting screws to support the posterior facet as well as using rafting screws with a larger diameter.

To perform the PACO technique, the surgeon needs to be skilled in hindfoot arthroscopy. Orthopedic surgeons with broad experience in both foot and ankle arthroscopy and traumatology will have advantages in learning and performing this technique. However, the fact that many departments that are not familiar with hindfoot arthroscopy treat this fracture implicates the limitations of the PACO technique in becoming the preferred surgical technique among most surgeons treating calcaneal fractures.

The strengths of our study are that all of the patients were operated upon with a standardized technique and have undergone a standardized rehabilitation protocol. The number of patients might seem low, but our cohort is one of the largest reporting outcomes after using the PACO technique.

The present study has some inherent weaknesses. It is retrospective and does not have a control group. There might have been a selection bias since the indication for using the PACO technique was made by the treating surgeon. Postoperative computed tomography was not obtained in all patients, which would have been of great value in evaluating the reduction of the posterior facet of the subtalar joint. Considering that 96% of our patients had subtalar osteoarthritis of at least a minimal grade, the clinical midterm results may worsen over time as subtalar osteoarthritis may advance. Long-term follow-up of our patients is necessary to evaluate further development of subtalar osteoarthritis and how this may affect the clinical outcome scores.

## Conclusion

The results in the present study suggest that percutaneous and arthroscopically assisted osteosynthesis of calcaneal fractures provides good clinical results with a low rate of surgical site infections and soft tissue complications. Prospective long-term studies will be necessary to better document the potential advantages and limitations of this operating technique.

## Supplemental Material

FAI914856_ICMJE – Supplemental material for Results After Percutaneous and Arthroscopically Assisted Osteosynthesis of Calcaneal Fractures Click here for additional data file. Supplemental material, FAI914856_ICMJE for Results After Percutaneous and Arthroscopically Assisted Osteosynthesis of Calcaneal Fractures by Wolfram Grün, Marius Molund, Fredrik Nilsen and Are Haukåen Stødle in Foot & Ankle International
